# Anxiety and stress in the postpartum: Is there more to postnatal distress than depression?

**DOI:** 10.1186/1471-244X-6-12

**Published:** 2006-03-24

**Authors:** Renée L Miller, Julie F Pallant, Lisa M Negri

**Affiliations:** 1 Faculty of Life and Social Sciences, Swinburne University of Technology, PO Box 218, Hawthorn, Victoria, 3122, Australia; 2 School of Health Sciences, RMIT University, PO Box 71, Bundoora, Victoria, 3083, Australia

## Abstract

**Background:**

Postnatal depression has received considerable research and clinical attention, however anxiety and stress in the postpartum has been relatively ignored. Along with the widespread use of the Edinburgh Postnatal Depression Scale (EPDS), depression has become the marker for postnatal maladjustment. Symptoms of anxiety tend to be subsumed within diagnoses of depression, which can result in anxiety being minimized or overlooked in the absence of depression. Some researchers have identified the need to distinguish between postnatal depression and anxiety, and to discern cases where depression and anxiety co-exist. The aim of this study was to assess the prevalence of postnatal distress using the EPDS and the Depression Anxiety Stress Scales (DASS-21).

**Method:**

As part of a larger cross-sectional study, the EPDS and DASS-21 were administered to a convenience sample of 325 primiparous mothers, who ranged in age from 18 to 44 years (M = 32 years). Recruited through mother's groups and health centres in Melbourne Australia, inclusion was limited to mothers whose babies were aged between 6 weeks and 6 months. Analyses included comparisons between the classifications of women according to the EPDS and the DASS-21, and an exploration of the extent to which the EPDS identified anxious-depressed women.

**Results:**

The EPDS identified 80 women (25%) as possibly depressed (using a cut-off of over 9), of which the DASS-21 corroborated 58%. In the total sample, 61 women (19%) were classified by the DASS-21 to be depressed. Using broader criteria for distress, it was revealed by the DASS-21 that a further 33 women (10%) showed symptoms of anxiety and stress without depression. A total of 41 women (13%) had symptoms of anxiety either in isolation or in combination with depression. The DASS-21 identified 7% of the sample as being both anxious *and *depressed. This at-risk sub-group had higher mean EPDS and DASS-depression scores than their depressed-only counterparts.

**Conclusion:**

The prevalence of anxiety and stress in the present study points to the importance of assessing postnatal women for broader indicators of psychological morbidity than that of depression alone. The DASS-21 appears to be a useful instrument for this purpose.

## Background

Although new motherhood is generally regarded as a joyous and meaningful experience, this period of transition involves a multitude of abrupt changes, and is recognized as a stressful life event [1-4]. It has been reported that around 13% of childbearing women suffer from postnatal depression (see meta-analysis by O'Hara & Swain [5]). The ramifications of undetected or untreated postnatal depression can be far-reaching for the mother, her infant, and her relationship with her partner [6-8].

Postnatal depression is the most prevalent of the postpartum mood disorders, and has therefore received the predominance of research attention in recent years (see Pope [9] for a review). However, several authors have pointed to the importance of distinguishing between postnatal depression and postnatal anxiety [7,9-11]. It is widely acknowledged that anxiety and depression (both as affective states and as clinical disorders) commonly co-exist [7,12]. This is also the case in the postpartum [13-16]. However, anxiety in the postpartum is generally subsumed within diagnoses of depression [9,17] as shown by the widespread use of the Edinburgh Postnatal Depression Scale (EPDS) in postnatal research [7]. Green [14] asserted that the concept of depression might in fact limit our understanding of postnatal distress, suggesting that broader indicators of negative mood need to be considered.

Milgrom, et al. [15] pointed to the importance of distinguishing anxiety from depression in order to provide appropriate treatments that target the symptoms and aetiology of anxiety specifically. This notion is supported by a study by Matthey et al. [7] that found that not all anxious mothers were depressed. Moreover, research with non-postpartum populations has shown that comorbidity of anxiety and depression – *anxious depression *– manifests more severe symptoms [18], is more difficult to treat than each disorder alone [19], is associated with poorer acute and long-term outcome [18], increases the risk for suicide [20], and requires specific treatment strategies for both sets of symptoms [19]. Matthey, et al. [7] pointed to the "hierarchical diagnostic custom" that requires depression to take precedence even when anxiety symptoms are a prominent feature (p. 144). This focus on depression can result in cases of anxiety (without depression) being undetected, and untreated. Similarly, in cases where depression and anxiety co-occur (*anxious-depression*), there is a risk that treatment strategies focus on the depressive symptoms, at the preclusion of specific treatments for the symptoms of anxiety. Ross, et al. [16] noted the importance of determining whether anxiety symptoms are part of a primary depression, or whether they form their own clinical entity.

Heron, et al. [11] suggested that anxiety may be a precursor to depression as a result of altered physiological pathways, or from the consequences of failing to manage stress. According to Lovibond and Lovibond [21], stress is a distinct negative emotional state that involves chronic arousal and impaired function. In a sample of new mothers, Terry, et al. [4] found evidence linking levels of stress and coping responses to depressive symptomatology.

### Measurement of postnatal depression

Several depression rating scales have been developed and validated on non-postpartum populations. However, the validity of these scales has been questioned in terms of their use in the postpartum, because some items (eg. lack of sleep, listlessness, weight loss, and poor concentration) are viewed as part of the 'normal' realm of being a nursing mother, rather than being due to depression [15,22]. Scales that include such symptoms may artificially inflate women's scores on depression, increasing the risk of false positives.

As a result of the potential confounding factors of some depression scales for the postpartum, the Edinburgh Postnatal Depression Scale (EPDS) was developed in 1987 by Cox, Holden, & Sagovsky [23]. The EPDS has been validated in numerous studies (see Pope [9] for a review), and has proven to be a simple, user-friendly, and reliable instrument for screening women for postnatal depression [22-24] in both clinical practice [15,25-29] and in research [30-34]. Although originally designed to measure depression, it has been suggested in a number of recent factor analytic studies [16,35] that the EPDS also taps into symptoms of anxiety.

Most studies use an EPDS score of over 12, which suggests a likelihood of depression [9]. However some researchers suggest a lower cut-off of over 9 to more sensitively detect women with depressive symptomatology [36]. Irrespective of the cut-off used, a positive identification on the EPDS flags the need for further assessment. Because the EPDS does not discriminate levels of depression, additional information is required to meet diagnostic criteria for depression. Several studies have used the Beck Depression Inventory (BDI), in conjunction with the EPDS, for the purposes of assessing the severity of depression in postnatal women [9]. Although it has been demonstrated that the BDI is a psychometrically robust instrument, its use in the postpartum calls for careful interpretation [37]. It has been noted that somatic symptoms that reflect the normal aspects of postnatal life (eg. lack of sleep, tiredness, and weight change) may inflate depression scores on the BDI [15,37], and that episodes of mild depression may not be detected by the BDI at the subclinical level in postnatal women [37].

### The Depression Anxiety and Stress Scales (DASS)

Empirical analyses in both non-clinical and clinical samples, have shown that conventionally regarded core symptoms of depression, such as sleep disturbance, changes in appetite, weight change, and loss of libido, are weak markers for the syndrome of depression [21]. Following extensive psychometric evaluations to maximally discriminate between depression and anxiety, Lovibond and Lovibond's 'Depression, Anxiety and Stress Scales' (DASS) [38], rejected items that have been identified as potential confounders within the BDI for use in the postpartum. These items included disturbance of sleep, appetite/weight loss, tiredness, lack of energy, and poor concentration [38]. Scale development of the DASS also yielded a third group of items that characterized a chronic non-specific arousal that the authors termed "stress" [38,39]. The DASS has been found to reliably distinguish between the symptoms of depression (dysphoric mood), anxiety (physiological arousal), and stress (psychological tension and agitation) [40], in both non-clinical [38-40] and clinical samples [40,41]. The authors recommend the use of the DASS in combination with other diagnostic information (such as clinical interviews).

### Broadening the criteria for postnatal distress

The postpartum period arguably represents one of the most important life stages for which the accurate detection and treatment of psychological distress is required. The transition to new motherhood has been associated with emotional distress in up to 30% of women [42], however depression has 'trumped' in terms of representing the diagnostic benchmark for postnatal maladjustment [7]. Given the potential for untreated postnatal distress to adversely affect the ongoing well-being of the mother and her infant, it seems pertinent to distinguish women's negative emotional states, in order to appropriately treat their unique symptoms. In order to represent a more complete picture of affective disturbance following childbirth, we suggest that the term *postnatal distress *be used to identify not only depression, but anxiety, and stress. The DASS was selected in the present study for a number of reasons: (1) its ability to identify these three negative emotional states as separate phenomena, (2) its' capacity to identify comorbidity of these negative emotional states, (3) the fact that it does not include the potential confounding factors for which other depression-severity scales have been criticized, and (4) its ability to identify mild symptoms of each negative affective state, in order to more fully identify women who might be distressed.

### Aims of the present study

According to Lovibond and Lovibond [21], stressful life events have the potential to precipitate episodes of anxiety and depression, and to "lead to a characteristic stress response involving chronic arousal and impaired function" (p. 335). Given that new motherhood holds the potential to be a stressful life event [2-4], and to elicit various cognitive-affective symptoms [9], it seems reasonable to propose that the DASS be used to assess the negative emotional states of, not only depression, but of anxiety and stress in postnatal women.

The primary aims of the present study were to assess the prevalence of *postnatal distress *in a sample of postnatal women, and to provide a preliminary investigation into the potential use of the EPDS in conjunction with the DASS. To this end, both the EPDS and the DASS were administered to this population. Firstly, the EPDS and the DASS depression scales were used to reflect the prevalence of postnatal depression in the total sample, and the extent to which these two scales corroborated their classifications. Secondly the prevalence of anxiety and stress was explored to form the basis of a broader concept of *postnatal distress*. Using a conceptualization of *postnatal distress *to include depression, anxiety and stress, the present study assessed the extent to which *anxious *and *stressed *mothers might have otherwise been missed using criteria for depression only (using both the EPDS and the DASS). Thirdly, the comorbidity of depression, and anxiety was examined, with particular interest in the sub-group of women who had a combination of depression and anxiety (*anxious-depression*) and the extent to which the EPDS detected these cases.

## Methods

### Participants

As part of a larger cross-sectional study (on first-time mothers' experiences of motherhood), a convenience sample was obtained in Melbourne, Australia, by inviting primiparous women to voluntarily participate in the study. The study was entitled "Factors associated with first-time mothers' experience of motherhood". Information posters, along with copies of the questionnaires (with reply paid envelopes), were placed in maternal and child health centres, mothers' groups, and doctors' waiting rooms. Women were invited to complete a questionnaire and return it via post, with no identifying information included. In order to reduce the potential confounds of additional children, criteria for inclusion limited participants to first time mothers with no step or foster children. Similarly, to reduce the variability of different stressors impacting women at later stages of infant development, participants were required to be between 6 weeks and 6 months postnatal. This time-period was also consistent with other research that has found that half the cases of postnatal depression occur in the first three months, and three quarters of cases by six months postpartum [43]. The study was approved by the Swinburne University of Technology Ethics Committee.

A total of 325 women participated in this study ranging in age from 18 to 44 years, with a mean age of 32 years (SD = 4.6). The age of women's babies at the time of completing the questionnaire ranged from 6 weeks to 6 months, with a mean age of 13 weeks (SD = 5.0). The majority of women (94%) were married (n = 248) or in a defacto relationship (n = 59), with 9 women (2.8%) in a non-cohabiting relationship, 5 women (1.5%) who were single, 2 women (0.6%) who were divorced, and 1 woman (0.3%) who was widowed. In relation to educational level, 103 women (31.9%) reported having had no tertiary education, 107 women (33.1%) reported having completed undergraduate university degrees, and 113 women (34.8%) reported having completed postgraduate university degrees.

### Measures

#### Depression Anxiety Stress Scales (DASS-21)

The DASS consists of three self-report scales that have been designed to measure the negative emotional states of depression, anxiety and stress [38]. Lovibond and Lovibond [38] reported that the depression scale measures "...dysphoria, hopelessness, devaluation of life, self-deprecation, lack of interest/involvement, anhedonia, and inertia"; the anxiety scale measures "autonomic arousal, skeletal muscle effects, situational anxiety, and subjective experience of anxious affect"; and the stress scale measures "difficulty relaxing, nervous arousal, and being easily upset/agitated, irritable/over-reactive and impatient" (p.1).

Initially normed on first-year university students [38], the DASS has also been normed on clinical samples revealing adequate psychometric properties [21]. Exploratory factor analyses showed a first order three-factor structure of depression, anxiety and stress [38,41]. Convergent and divergent validity for the DASS has been demonstrated whereby the DASS depression scale correlated strongly with the BDI-II (r = .74), and the DASS anxiety scale was highly correlated with the Beck Anxiety Inventory (BAI) (r = .81) [21,38].

The DASS-21 is a brief 21 item version of the full DASS, which originally consisted of 42 items. Each of the three DASS-21 scales contains seven items representing the dimensions of depression, anxiety and stress. Participants are asked to rate the extent to which they experienced each state over the past week on a 4-point likert rating scale. Sub-scale scores are derived by totaling the scores. No items are reverse scored. Scores for each sub-scale are multiplied by two to ensure consistent interpretation with the longer 42 item version [38]. The DASS manual [38] provides a series of cut-off values to classify individuals into severity rating categories. These severity ratings are based on percentile scores, *with 0–78 classified as 'normal', 78–87 as 'mild', 87–95 as 'moderate', 95–98 as 'severe', and 98–100 as 'extremely severe' *[38]. Lovibond and Lovibond's [38] reported alpha values for the DASS-21 from a student sample (N = 717) were .81 for depression, .73 for anxiety, and .81 for stress. In a clinical sample, Clara, Cox, and Enns [44] reported high levels of internal consistency for the DASS-21 with alpha values of .92 for depression, .81 for anxiety, and .88 for stress. Internal consistency in the present study was explored for each scale of the DASS-21, and Cronbach alpha coefficients were adequate: Depression (.84), Anxiety (.77) and Stress (.86).

#### Edinburgh Postnatal Depression Scale (EPDS)

The EPDS has been designed to screen postnatal women for the likelihood of postnatal depression [23]. The 10-item scale assesses symptoms of anhedonia and reactivity, self-blame, anxiety, panic, coping, insomnia (due to unhappiness), sadness, tearfulness and self-harm [22]. The EPDS excludes somatic symptoms such as fatigue and change in appetite, which may occur normally in the postpartum, and which may otherwise potentially not discriminate depressed from non-depressed women.

This 10-item scale includes questions such as "*I have been able to laugh and see the funny side of things*" and "*I have blamed myself unnecessarily when things went wrong*". Respondents indicate on a four-point likert scale, the response that best describes the way they have been feeling over the past seven days. Items are scored from 0 to 3 with a maximum total score of 30. The EPDS has been used extensively as a screening tool in clinical practice and in research into various aspects of postnatal depression [9]. Good psychometric properties, including sensitivity and specificity, have been reported for the EPDS using a cut-off of 12 [20,22,23,45]. Some authors have recommended a cut-off of over 9 on the EPDS to increase its' sensitivity in community screening [34,46], despite the fact that many of the women scoring above this threshold may not meet the criteria for clinical depression. This lower threshold has resulted in reported sensitivity values of 84–100% and specificity values of 82–88% [45,46]. The reliability and validity of the EPDS has been well-documented [37]. Cronbach's alpha for the EPDS in the present study was .88.

## Results

### Prevalence of depression according to the EPDS and the DASS

In the present sample of postnatal women (N = 325), the mean score on the EPDS was 6.94 (SD = 4.8). The EPDS identified 36 women (11.3%) to have a score above the commonly recommended cut-off of 12 [23]. However, using a more sensitive cut-off of 9 [34,46] 80 women (25%), were identified as *likely *to be *depressed*. From this point on in the results section, women identified as likely to be depressed using a cut-off of over 9 on the EPDS, will be referred to as *likely depressed*. Those women scoring 9 or less on the EPDS will be referred to as *unlikely depressed*.

Consistent with common clinical practice, additional information is required to assess the severity of depression for the women identified as *likely depressed *on the EPDS. In this study the DASS-21 depression scale was used for this purpose. The mean DASS-21 depression score was 5.10 (SD = 6.4), the mean DASS-21 anxiety score was 3.33 (SD = 5.3) and the mean DASS-21 stress score was 10 (SD = 8.1). The recommended cut-offs in the DASS manual [38] were utilized, resulting in five severity categories: *normal*, *mild*, *moderate*, *severe*, and *extremely severe*. Women scoring in the *normal *category on depression were referred to from this point on as *non-depressed*, and women scoring in the *mild*, *moderate, severe *and *extremely severe *categories were referred to as *depressed*. On the DASS-21, the number and percentage of women identified in this sample as *depressed *was 61 (19%).

**Figure 1:**
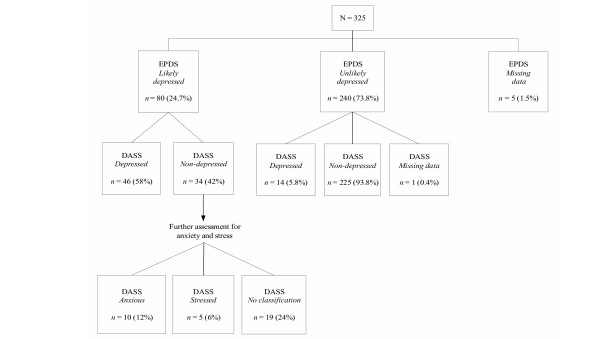
DASS-21 classifications for women identified as depressed on the EPDS.

An exploration was conducted of the two depression measures in terms of cross classifications. These findings are presented in Figure 1.

### Prevalence of postnatal distress according to the DASS, and the EPDS

The present study proposed a broader classification for assessing postnatal *distress*, over and above that of the usual focus on depression. To this end, the DASS-21 was used to assess the severity levels of not only depression, but of anxiety and stress as well. According to the recommended cut-offs in the DASS manual [38] scores were classified into severity categories of *normal*, *mild*, *moderate*, *severe*, and *extremely severe*, with respect to depression, anxiety and stress. Women who scored in the *normal *range on depression, anxiety or stress, are referred to here as *non-depressed*, *non-anxious *and *non-stressed *respectively. Women who scored in the *mild *to *extremely severe *ranges, were referred to as *depressed*, *anxious *or *stressed *respectively.

The first step was to investigate the 80 women who were identified by the EPDS as *likely depressed*, and to present the corresponding DASS-21 classifications for these women. Figure 1 presents the typical clinical pathway, whereby once the 80 women had been identified as being likely candidates for depression (on the EPDS), these women would have been assessed further. According to the DASS-21, 46 women were classified as *depressed*, and 34 were not. Applying the broader criteria of anxiety and stress using the DASS-21, it was revealed that of the 34 women identified by the EPDS as *likely depressed*, but not corroborated by the DASS-21 depression scale as *depressed*, 10 women were identified as *anxious*, and 5 women as *stressed*. Nineteen women received no classification on the DASS-21 (see Figure 1).

**Figure 2:**
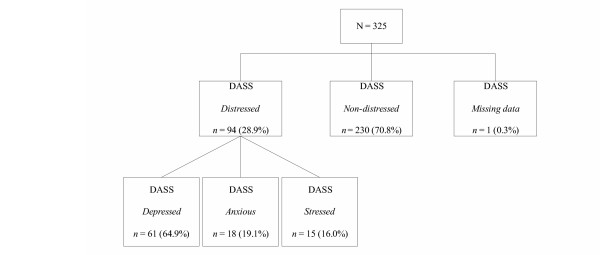
Classification of women on the DASS-21.

The next step was to investigate the prevalence of depression, anxiety and stress for the total sample, using the DASS-21. Women who scored in the *mild, moderate*, *severe *and *extremely severe *categories on at least one DASS-21 scale (depression, anxiety, or stress) were considered to be postnatally-*distressed*. Using these criteria, 94 women (29%) in the total sample were identified as postnatally-*distressed*. Figure 2 presents the number and percentages of women who were *depressed*, *anxious *and *stressed *in the total sample.

According to the DASS-21, an additional 33 women (10%) over and above the 61 women (19%) identified as *depressed *in the total sample, were classified as postnatally *distressed *(i.e., with classifications of anxiety and/or stress). In other words, had depression been the only criterion for which the present sample had been assessed, 33 *distressed *women (a further 10% of the total sample) would have been missed using the DASS-21 depression scale alone.

Returning to the EPDS classifications, of the 80 women identified by the EPDS as *likely depressed*, 46 women (58%) were correspondingly identified as *depressed *using the DASS-21 depression scale. However of these 80 EPDS-identified women, 15 (19%) were not corroborated by the DASS-21 as being *depressed*, which may have resulted in no further assessment if depression had been the sole criteria. With the inclusion of the DASS-21 anxiety and stress scales, these 15 *distressed *women were able to be identified, whereby 10 women were found to be *anxious*, and 5 women were identified as *stressed*. Of the 240 women classified by the EPDS as *not likely to be depressed*, the DASS-21 revealed that 14 women (6%) were classified as *depressed*. Similarly, of the 240 women identified by the EPDS as *not likely to be depressed*, 8 women (3%) were *anxious*, and 10 women (4%) were *stressed*.

These findings indicate that despite the sensitive EPDS cut-off of over 9, 14 *depressed *women were not identified by the EPDS as *likely to be depressed*, and 18 women who were postnatally *distressed *would not have been assessed further, had the EPDS been the initial screening tool used. With respect to the presence of anxiety in the total sample, 41 women (13%) had symptoms of anxiety either in isolation or in combination with depression and/or stress. This finding further supports the need to include anxiety in the assessment of postnatal women.

### Comorbidity of depression, anxiety and stress

**Figure 3:**
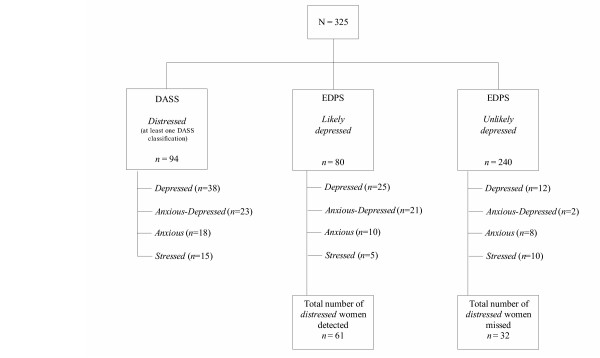
Classification of women on both the DASS-21 and EPDS.

Of particular interest in the present study was the sub-group of women who had both symptoms of depression and of anxiety (*anxious-depression*). Figure 3 shows the classifications for women in the present sample, according to both EPDS and the DASS-21, with *anxious-depressed *women reflected as a separate group from the women who were just *depressed*.

Figure 3 shows that out of the total sample of women, the DASS-21 found 38 women (12%) to be *depressed*, 23 women (7%) to be *anxious-depressed*, 18 women (6%) to be *anxious *(without depression), and 15 women (5%) to be *stressed *(without depression or anxiety). An interesting finding was the capacity of the EPDS to detect *anxious-depressed *women more accurately than it failed to detect *anxious-depressed *women. In order to explore this finding statistically, four groups of women were generated according to their classification on the DASS-21. This was done to determine whether there were differences among these four groups of women on EPDS scores. The four groups were the *depressed *group (n = 37), the *anxious-depressed *group (n = 23), the *anxious *group (n = 18) and the *stressed *group (n = 15). Because of the negative skew in the distribution of scores on the EPDS (the dependent variable), a non-parametric Kruskal-Wallis test was used. Results indicated a statistically significant difference among the groups. Inspection of the mean ranks revealed that the highest scores on the EPDS were found for the *anxious-depressed *group (mean rank = 70.83), followed by the *depressed *group (mean rank = 44.35), the *anxious *group (mean rank = 38.69) and the *stressed *group (mean rank = 26.97) (chi square = 28.42, df = 3, p < .001). These findings point to the capacity of the EPDS to detect *anxious-depression*.

In order to further interpret these findings, the DASS-21 severity categories (i.e., *mild*, *moderate*, *severe*, and *extremely severe*) were explored for each defined group of women (i.e., *depressed*, *anxious-depressed*, *anxious*, and *stressed*), with the corresponding number of women who received an EPDS classification (i.e., *likely depressed *and *unlikely depressed*) (see Table 1).

**Table 1:** Numbers of women in each scoring category on DASS-21 depression, anxiety and stress scales for each classified group (*depressed*, *anxious-depressed*, *anxious*, and *stressed*), and the number of women with corresponding EPDS identifications

	*Mild*	*Moderate*	*Severe*	*Extremely Severe*	*No EPDS identification (*i.e., *unlikely depressed*)
***Depressed *group **(N = 38)					
on DASS-depression	24	12	1	1	
on EPDS-*likely depressed*	14	9^a^	1	1	12
					
***Anxious-depressed *group **(N = 23)					
on DASS-depression	3	9	9	2	
on EPDS-*likely depressed*	3	7	9	2	2^b^
on DASS-anxiety	5	8	3	7	
on EPDS-*likely depressed*	4	8	2	7	2^b^
					
***Anxious *group **(N = 18)					
on DASS-anxiety	10	4	1	3	
on EPDS-*likely depressed*	5	1	1	3	8
					
***Stressed *group **(N = 15)					
on DASS-stress	8	4	3		
on EPDS-*likely depressed*	2	1	2		10

^a ^Missing data for one woman who failed to complete the EPDS.

^b ^The EPDS missed 4 classifications, 2 on depression and 2 on anxiety, which accounted for 2 cases of *anxious-depression*.

The most notable findings in Table 1 are that the EPDS, despite the sensitive cut-off of over 9, did not detect 10 women who had *mild *symptoms of depression, and 2 women who had *moderate *symptoms of depression. The EPDS identified almost all of the *anxious-depressed *women, except for 2 women, both of whom had DASS-21 scores that were *moderate *on depression and *severe *on anxiety. Interestingly, the EPDS (which is a tool to detect the likelihood of depression) identified 10 out of 18 women who were anxious (but not depressed). Similarly, the EPDS detected 5 cases of *stress *(without depression). Inspection of Table 1 suggested that the *anxious-depressed *group of women had more severe scores on DASS-21 depression than the women who were *depressed *(without anxiety). In order to explore this statistically, a Mann-Whitney U test was conducted to determine whether there was a difference between these two groups on DASS-21 depression scores. A significant difference was found between these two groups whereby the *anxious-depressed *group (mean rank = 42.02) had higher scores on DASS-21 depression than the *depressed *(only) group (mean rank = 24.33: Z=-3.84, p < .001).

## Discussion

The present study proposed a broader classification for postnatal *distress*, to include measures of not only depression, but of anxiety and stress as well. Some authors have suggested that many women experience distress in the postpartum that is potentially missed using the criteria for depression alone [7,17]. These authors have stated that it is incorrect to conclude that women are functioning well just because they do not fit the criteria for depression. This notion is particularly relevant in light of the potential adverse consequences of untreated distress in new mothers at this critical time.

Applying a broader conceptualization of *postnatal distress *in the present study, 94 women (29% of the total sample) were found to have at least one classification of depression, anxiety or stress, in the *mild*, *moderate*, *severe *or *extremely severe *categories on the DASS-21 [38]. This finding is consistent with Johnson et al. [42], who reported that 30% of newly delivered women were emotionally distressed. The EPDS identified 61 of the 94 distressed women as *likely depressed*, although according to the DASS-21, 15 of these women were not *depressed*, but were rather *anxious *and/or *stressed*. As is common practice with the use of the EPDS as a screening tool, an index of the severity of depression is required. However, in practice, if a depression scale (in this case the DASS-21 depression scale) had been the only measure used to assess the severity of the 80 EPDS-identified women, these 15 *non-depressed*–but clearly, *distressed*–women would have potentially been overlooked for further investigation and assistance.

The EPDS identified 10 out of 18 *anxious *women, and 21 out of 23 *anxious-depressed *women, showing support for the previous research findings concerning an anxiety subscale within the EPDS [16,35,47-49]. The EPDS however, did not identify 14 women (4%) who were found by the DASS to have *mild *and *moderate *symptoms of depression. This is surprising given the sensitive cut-off (of over 9) that was used on the EPDS. However, with the capacity of the DASS to detect *mild *cases of depression, these women, in practice, could have been attended to, and followed up for worsening symptomatology by the application of the DASS-21 over time. Of more concern, is that the EPDS, as a screening tool, did not identify 18 women (6%) who were *distressed *(i.e, *anxious *and/or *stressed *on the DASS-21). The implication for these 18 women is that in practice, they could 'fall through the cracks' due to an over-reliance on depression being the marker for distress in the postpartum. Taken together, the findings of this study demonstrate that a total of 33 women (10% of the total sample) might have been overlooked if depression had been the sole marker upon which postnatal distress was determined.

The present study not only showed the ability of the DASS-21 to disentangle classifications of depression, from anxiety and stress, but also demonstrated its capacity to identify comorbid classifications. The sub-group of *anxious-depressed *women was of particular interest in the present study as some authors have identified that (non-postpartum) patients in whom anxiety and depression co-occur, manifest more severe symptoms [18] are more difficult to treat [19], show poorer acute and long-term outcomes [18], and are at increased risk for suicide [20] than patients with either pure anxiety or depression. It has also been reported that patients with sub-threshold depression and anxiety are at a greater risk for developing a threshold disorder following a psychosocial stressor [50]. In the present study, the DASS-21 identified 61 women (19%) to be *depressed*, of whom 23 (7% of the total sample) were also *anxious *(*anxious-depressed*). These *anxious-depressed *mothers were found to have significantly higher levels of depressive symptoms on both the DASS-21 and the EPDS compared to participants who reached criteria for depression alone. These findings point to the clinical importance of detecting this group so that appropriate interventions can be implemented.

Arguably, new motherhood is one of the most important life stages requiring the accurate detection and treatment of distress. The findings of the present study demonstrate the utility of the DASS-21, in conjunction with the EPDS, for detecting depression, anxiety and stress as distinct emotional states; for detecting cases where a combination of symptoms are present; and for its capacity to detect mild cases of depression, with reduced likelihood of confounding depression with normal concomitants of postpartum life.

### Limitations of the present study

There are a number of limitations of the study that may restrict its generalizability. Respondents were recruited from mothers' groups; health centres and doctors' surgeries by placing posters encouraging women to collect and complete a questionnaire concerning their experience of first time motherhood. Individual women were not approached directly. Given the non-intrusive data collection procedure used in this study there was no way of assessing the representativeness of the women choosing to volunteer. The high proportion of tertiary educated women (i.e., two thirds of the sample) may be a reflection of a self-selection bias, with highly educated women being more willing to take the time to complete and return the questionnaire. Further research is needed to replicate this study using sampling designs that allow a more representative sample of women from a wider variety of backgrounds, and socio-economic and educational levels. No formal diagnostic structured interview was conducted in this study in order to provide a 'gold standard' against which to validate the DASS-21 for its use in postnatal populations. Further studies are necessary to address this issue and to also assess the usefulness of the DASS-21 in populations of antenatal women [10,14,16] and multiparous women.

## Conclusion

The present study points to the need for a broader assessment of distress in the postpartum with a view to prevention and early intervention [10]. The DASS-21 appears to be a useful tool for broadening the criteria for postnatal *distress*, over and above that of depression to include anxiety and stress. It may serve as a worthwhile addition to the widely used EPDS, by providing continuous ratings of severity on the three negative emotional states of depression, anxiety, and stress, and by identifying the comorbidity of these states. For as long as the focus of postnatal maladjustment is on depression, failure to identify and treat significant symptoms of anxiety and stress may leave women vulnerable to worsening symptomatology. Untreated maternal distress can have a substantial impact on the well-being of the mother, her relationships and her infant. The DASS-21 is a brief, easy to administer, inventory that may assist practitioners (along with clinical interviews) to more effectively assess and treat new mothers who may be depressed, anxious and/or stressed in the postpartum.

## Competing interests

The author(s) declare that they have no competing interests.

## Authors' contributions

JP supervised the design of the study and the statistical analyses undertaken. RM collected and analyzed the data and prepared a draft of the article. LN advised on the research design and preparation of the article. All authors contributed to the preparation of the article. All authors read and approved the final manuscript.

## Pre-publication history

The pre-publication history for this paper can be accessed here:


